# Effect of energy back transfer from Er^3+^ to Yb^3+^ ions on the upconversion luminescence of Er:NaYb(MoO_4_)_2_ and Yb,Er:NaBi(MoO_4_)_2_

**DOI:** 10.1007/s12200-025-00155-5

**Published:** 2025-05-15

**Authors:** Miaomiao Wang, Mengyu Zhang, Shoujun Ding, Haitang Hu, Chuancheng Zhang, Yong Zou

**Affiliations:** 1 School of Microelectronics and Data Science, Anhui University of Technology, Maanshan, 243002 China; 2 Anhui Provincial Joint Key Laboratory of Disciplines for Industrial Big Data Analysis and Intelligent Decision, Maanshan, 243002 China

**Keywords:** Upconversion luminescence, Energy back transfer, Yb^3+^/Er^3+^ cooping, Phosphor, Crystal

## Abstract

Under the excitation of a 980 nm laser, the visible upconversion (UC) luminescence of Er^3+^ ions doped Yb^3+^ ions self-activated NaYb(MoO_4_)_2_ phosphor and crystal, as well as the Yb^3+^/Er^3+^ ions codoped NaBi(MoO_4_)_2_ crystal were investigated comprehensively. The results indicate that all three samples exhibit two significant green emission bands and a weak red emission band in the visible band corresponding to the transitions of ^2^H_11/2_/^4^S_3/2_ → ^4^I_15/2_ and ^4^F_9/2_ → ^4^I_15/2_ of Er^3+^ ions, respectively. Through the variable power density spectra of three different samples, the relationship between the energy back transfer (EBT) process of Yb^3+^-Er^3+^ ions and the power density point and Yb^3+^ ion concentration was investigated. The EBT process was observed in both the Er^3+^ ions doped Yb^3+^ ions self-activated NaYb(MoO_4_)_2_ phosphor and crystal, as confirmed by the luminescence image of the sample. At high power density, the Yb^3+^ ions self-activated sample exhibited yellow luminescence, with the crystal appearing later than the phosphor. In contrast, the NaBi(MoO_4_)_2_ crystal displayed bright green emission within the measured power density range. In addition, by monitoring the relative intensity change of Yb^3+^ emission in 5 at% Er^3+^:NaYb(MoO_4_)_2_ crystal, the generation of EBT process in self-activated samples at high power density is more directly explained. These experimental results provide a reliable basis for our comprehensive understanding of the EBT mechanism, and also provide a reliable direction for the final determination of the optimal excitation power density for optical temperature measurement.

**Graphical Abstract:**

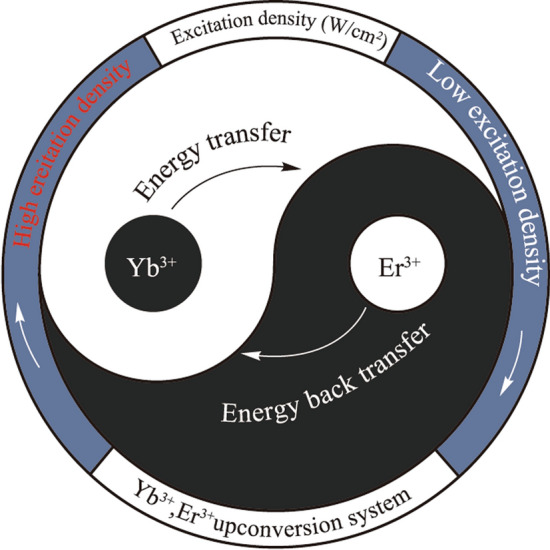

## Introduction

Upconversion (UC) luminescence is the process of achieving higher energy visible emission photons by continuously absorbing at least two low-energy photons [1]. Rare-earth ions doped UC luminescent materials have attracted the attention of many researchers, including potential applications in biological imaging, solid-state lasers, fluorescent anti-counterfeiting and temperature sensors [1–5]. Of particular interest is the optical temperature sensors based on fluorescence intensity ratio (FIR), which enable non-contact, high-precision and fast temperature measurement [6–8]. The FIR technology is based on the intensity ratio of emission bands, corresponding to the two thermally coupled energy levels of rare earth ions, to realize the detection of temperature-dependent UC luminescence intensity [8]. At present, numerous Yb^3+^ and Er^3+^ ions codoped temperature sensing luminescent materials based on FIR technology have been effectively synthesized, including NaGd(MoO_4_)_2_ [9], La_4_GeO_8_ [10], Y_2_O_3_ [11], Cs_2_NaGdCl_6_ [12], NaYF_4_ [13], among others. Unfortunately, we found that these researchers were not very comprehensive in their discourse on the mechanism of UC luminescence, and even some of the literature neglected to describe the energy back-transfer (EBT) mechanism.

Numerous scholars have extensively investigated the UC luminescent materials codoped with Yb^3+^ and Er^3+^ ions, with a discussion on the UC luminescence mechanism [14–16]. Among them, some literatures also mentioned the EBT mechanism. It is noteworthy that EBT is intricately linked to excitation power, but the excitation power used in the existing optical temperature measurement experiments is not consistent. For example, Chen et al. [10] focused on the optical temperature measurement of Y_2_GeO_5_:Er^3+^, Yb^3+^ phosphors, revealing a peak absolute sensitivity (*S*_A_) of 1.85 × 10^3^ K^−1^ at 473 K (excitation power: 150 mW). Similarly, Liao et al. [17] explored the optical temperature measurement of Gd_2_TiO_5_: 2 at% Yb^3+^/2 at% Er^3+^ phosphors, identifying a maximum *S*_A_ of 40.76 × 10^4^ K^−1^ at 565 K (excitation power density: 3 W/cm^2^). Additionally, Li et al. [18] observed that KBaY(MoO_4_)_3_:Yb^3+^, Er^3+^ phosphors exhibited a peak *S*_A_ of 0.01206 K^−1^ at 420 K (excitation power density: 4.6 W/cm^2^). EBT represents a crucial mechanism state affecting the layout of Yb^3+^/Er^3+^ ion intermediate states [3, 19]. Regrettably, current literature often only mentions the existence of this mechanism, yet fails to provide an in-depth analysis encompassing aspects such as sample conditions, doped Yb^3+^ ion concentrations, and the correlation between excitation power and EBT.

Consequently, to comprehensively investigate the process of EBT in the UC luminescence mechanism, various samples were synthesized with differing states (phosphors and crystals) and different doping conditions to conduct experiments. The experiment aimed to enhance the understanding of the EBT mechanism, and establish a pathway for the optimum excitation power point for optical temperature measurements. The characteristics of the EBT process between the phosphor (polycrystalline) and the crystal (monocrystalline), that is, the local order and long-range order systems, were analyzed and compared. The impact of Yb^3+^ ion concentration on the EBT process was scrutinized under various conditions including Er^3+^ ions doped Yb^3+^ ions self-activation and Yb^3+^/Er^3+^ ions codoping configurations.

## Experimental section

The NaYb(MoO_4_)_2_: 5 at% Er^3+^ phosphor was synthesized by high temperature solid state method, and the crystals of NaYb(MoO_4_)_2_: 5 at% Er^3+^ and NaBi(MoO_4_)_2_: 10 at% Yb^3+^, 1 at% Er^3+^ were successfully grown by Czochralski method using a JGD-400 top-mounted single crystal growth furnace (produced by CETC26 th) [20]. The UC emission spectra of the samples were measured using an Omni-*λ*5028i and a charge coupler (Andor DU401 BVF). The excitation source for the UC emission was a 980 nm semiconductor laser with a maximum output power of 10 W (BWT Beijing Ltd.).

The phase structural analysis was performed using a Bruker D8 Advance X-ray diffractometer equipped with Cu-Kα radiation (*λ* = 1.5406 Å). The diffraction angle (2*θ*) ranged from 10° to 80° with a step size of 0.02°. The diffuse reflectance UV–Vis–IR absorption spectra were measured on a UV-3600 spectrophotometer.

## Results and discussion

**Fig. 1:**
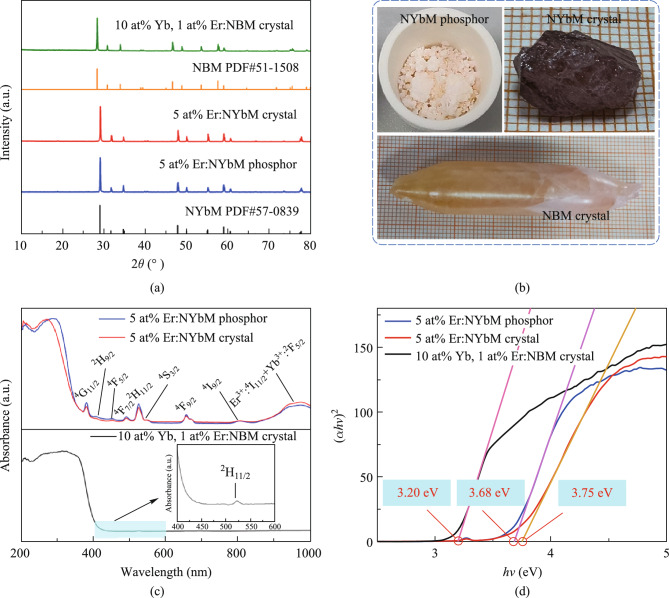
**a** XRD patterns measured for the 5 at% Er^3+^:NaYb(MoO_4_)_2_ phosphor, 5 at% Er^3+^:NaYb(MoO_4_)_2_ crystal, 10 at% Yb^3+^, 1 at% Er^3+^:NaBi(MoO_4_)_2_ crystal, together with the PDF#57–0839 (NaYb(MoO_4_)_2_) and PDF#51–1508 (NaBi(MoO_4_)_2_). **b** Photograph of all samples. **c** UV–Vis–IR diffuse reflectance spectra of all samples. **d** Tauc plots of (*αhν*)^2^ versus *hν* for all samples

Figure 1a displays the X-ray diffraction (XRD) patterns for the relevant phosphor and single crystals. All patterns correspond to the standard reference compounds NaYb(MoO_4_)_2_ (PDF#57–0839) and NaBi(MoO_4_)_2_ (PDF#51–1508), demonstrating the pure phase state of the prepared samples and that the crystal structure of the main lattice is not significantly altered by the addition of dopant ions. The photograph of the as-prepared samples is given in Fig. 1b.

Figure 1c shows the measured diffuse reflection spectra of Er^3+^ doped NaYb(MoO_4_)_2_ samples and Er^3+^/Yb^3+^ co-doped NaBi(MoO_4_)_2_ single crystal in the range of 200–1000 nm. In the Er^3+^ doped NaYb(MoO_4_)_2_ samples, nine absorption bands with considerable intensity were displayed, corresponding to the transition from the ^4^I_15/2_ ground state of Er^3+^ to different excited states. These absorption peaks are located at 379, 407, 451, 489, 523, 545, 655, 802, and 970 nm, corresponding to the transitions of the excited states ^4^G_11/2_, ^2^H_9/2_, ^4^F_5/2_, ^4^F_7/2_, ^2^H_11/2_, ^4^S_3/2_, ^4^F_9/2_, ^4^I_9/2_, and ^4^I_11/2_, respectively [21]. Among them, the absorption band appears wider near 970 nm, which may be due to the superposition of two absorption peaks, attributed to the ^4^I_15/2_ → ^4^I_11/2_ transition of Er^3+^ and the ^2^F_7/2_ → ^2^F_5/2_ transition of high concentration of Yb^3+^ ions in the host. In addition, an absorption band corresponding to the transition from the ground state ^4^I_15/2_ to the excited state ^2^H_11/2_ of Er^3+^ ions was observed in the Er^3+^/Yb^3+^ co-doped NaBi(MoO_4_)_2_ single crystal, and its absorption peak was at 523 nm.

The optical band gap energy (*E*_g_) of the samples is estimated from the diffuse reflectance spectra using the Tauc relation given in [22]

1 $${(\alpha h\nu )}^\frac{1}{n}=A\left(h\nu -{E}_{\text{g}}\right),$$

where $\alpha$ is the absorption constant, ℎ is Planck’s constant, $\nu$ is the frequency of the incident photon, *A* is the proportionality constant, *E*_g_ is the material band gap, the index *n* represents the transition properties that occur in the sample, and their values are *n* = 1/2 and 2 for direct and indirect transitions, respectively. Here, we consider the direct band gap, so the value of *n* remains 1/2. The Tauc plots of the prepared samples are shown in Fig. 1d. The band gap of Er^3+^/Yb^3+^ co-doped NaBi(MoO_4_)_2_ single crystal is observed to be 3.20 eV, while the band gap of Er^3+^ doped NaYb(MoO_4_)_2_ phosphor is calculated to be 3.68 eV. This is attributed to the larger ionic radius of Bi^3+^ ions, leading to a smaller band gap in the former [23]. In addition, the band gap of Er^3+^ doped NaYb(MoO_4_)_2_ single crystal is slightly larger than that of the phosphor. This is due to the segregation coefficient of Er^3+^ within the NaYb(MoO_4_)_2_ host, resulting in incomplete substitution of Yb^3+^ by doped Er^3+^ during crystal growth.

**Fig. 2:**
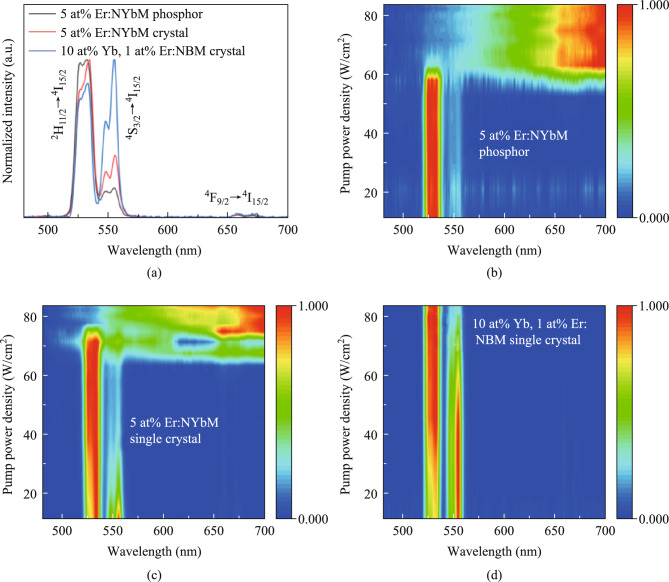
**a** Normalized emission spectra of 5 at% Er^3+^:NaYb(MoO_4_)_2_ phosphor, 5 at% Er^3+^:NaYb(MoO_4_)_2_ crystal, 10 at% Yb^3+^, 1 at% Er^3+^:NaBi(MoO_4_)_2_ crystal upon the excitation of 980 nm laser. The contour plots of **b** 5 at% Er^3+^:NaYb(MoO_4_)_2_ phosphor, **c** 5 at% Er^3+^:NaYb(MoO_4_)_2_ crystal, and **d** 10 at% Yb^3+^, 1 at% Er^3+^:NaBi(MoO_4_)_2_ crystal upon the excitation of 980 nm laser

To investigate the correlation between the sensitizer Yb^3+^ ions and the UC luminescence of the materials, the UC spectra of the three synthesized samples were measured under 980 nm laser excitation, as illustrated in Fig. 2a. The samples exhibited two prominent green emission bands and a weak red emission band within the visible light band. Among them, the green emission bands are a result of the transitions ^2^H_11/2_ → ^4^I_15/2_ (centered at 530 nm) and ^4^S_3/2_ → ^4^I_15/2_ (centered at 554 nm) involving the Er^3+^ ions. The weak red emission band originates from the transition ^4^F_9/2_ → ^4^I_15/2_ (centered at 660 nm) of the Er^3+^ ions. It is worth noting that the concentration of Yb^3+^ ions in the matrix does not exert a significant influence on the emission peak position of the UC emission spectra.

The contour plots for different power densities of three different samples were examined to learn more about the UC luminescence mechanism. The corresponding contour plots obtained after normalizing the data were shown in Fig. 2b–d. Over a broad power density range, the concentration of Yb^3+^ ions and the pump power density were examined in relation to the energy back transfer (EBT) process of Yb^3+^-Er^3+^ ions. A comparison of the three spectra reveals that the spectrum of the NaBi(MoO_4_)_2_ crystal remains constant throughout the power density range, dominated by the green emission peak. Conversely, the spectra of the self-activated NaYb(MoO_4_)_2_ phosphor and NaYb(MoO_4_)_2_ crystal were distorted at higher power density, potentially attributed to the emergence of the EBT process between Yb^3+^-Er^3+^ ions under high-power density conditions.

**Fig. 3:**
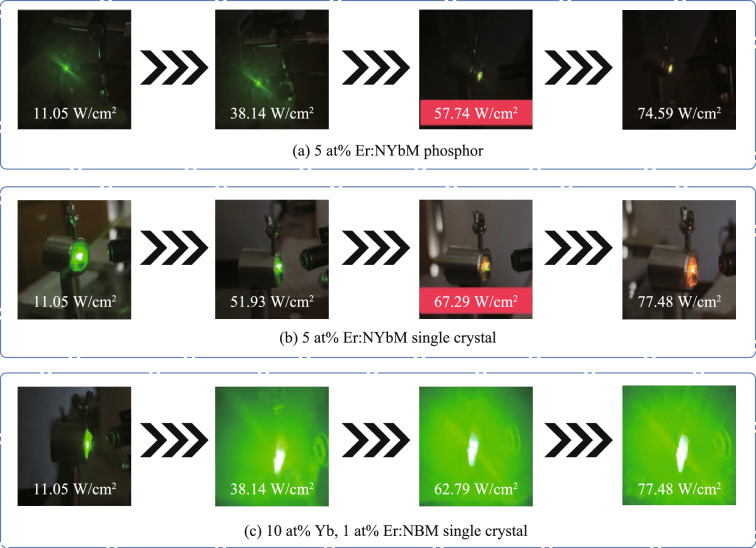
Luminescence images of **a** 5 at% Er^3+^:NaYb(MoO_4_)_2_ phosphor, **b** 5 at% Er^3+^:NaYb(MoO_4_)_2_ crystal, and **c** 10 at% Yb^3+^, 1 at% Er^3+^:NaBi(MoO_4_)_2_ crystal upon the excitation of 980 nm laser with different pump power density

In addition, to illustrate the phenomenon more intuitively, the luminescence images of the three samples were captured at different power densities, as shown in Fig. 3. The NaYb(MoO_4_)_2_ phosphor and crystal exhibit a yellow luminescence trend at higher power densities, and the phosphor has demonstrated yellow luminescence at lower power densities than the crystal. This may be due to the better thermal stability of the crystal compared to the phosphor, so that the power point at which its EBT occurs appears to be shifted back. The thermal conductivity of a material is intricately linked to its internal structure. In general, the long-range ordered structure will have better thermal conductivity. Thus, in contrast to phosphors exhibiting a locally ordered structure, crystals possessing a long-range ordered structure demonstrate better thermal stability, potentially accounting for the weaker EBT process within crystals.

Notably, the luminescence image of the Yb^3+^/Er^3+^ ions codoped NaBi(MoO_4_)_2_ crystal in Fig. 3c shows that the sample exhibits bright green emission throughout the power density range. This observation is consistent with the findings depicted in the contour plot of Fig. 2d, indicating the absence of an EBT process between Yb^3+^ and Er^3+^ ions. The results of the experiment revealed a direct correlation between the concentration of Yb^3+^ ions and the EBT process of Yb^3+^-Er^3+^ ions, with the excitation power density level also playing a role in this process. A higher concentration of Yb^3+^ ions was found to facilitate the EBT process between Yb^3+^ and Er^3+^ ions, leading to the suppression of green light emission and the enhancement of red light emission from Er^3+^ ions. Furthermore, it was observed that the phosphor initiated the EBT process at a lower power threshold compared to the crystal, which may be related to the poor thermal stability of the phosphor.

**Fig. 4:**
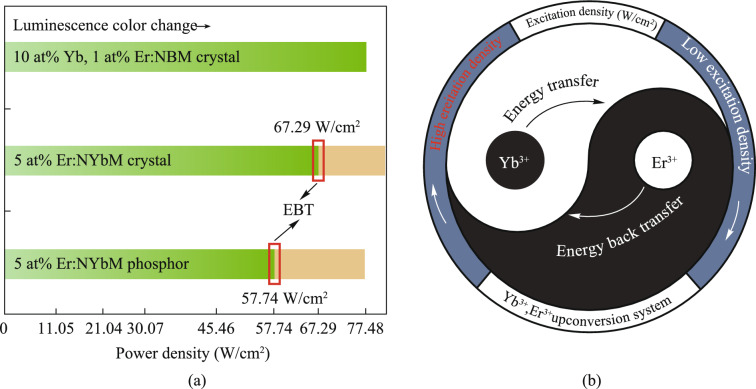
**a** Relationship between sample luminescence and power density as well as EBT process. **b** Schematic diagram of the energy transfer relationship between Yb^3+^ and Er^3+^ ions

Figure 4a summarizes the qualitative relationship between pump power density and luminescence color for the three samples. The NaYb(MoO_4_)_2_ phosphor exhibits the earliest transition to yellow emission (lower power threshold), followed by the NaYb(MoO_4_)_2_ crystal, while the NaBi(MoO_4_)_2_ crystal retains green emission throughout. This trend aligns with the thermal stability and Yb^3+^ concentration differences between samples. The color shift is attributed to EBT-driven suppression of green emission and enhancement of red emission, as confirmed by spectral distortion analysis (Fig. 2b–d) and luminescence imaging (Fig. 3). Furthermore, a schematic diagram of the energy transfer relationship between Yb^3+^ and Er^3+^ ions is provided to enhance the understanding, as depicted in Fig. 4b.

**Fig. 5:**
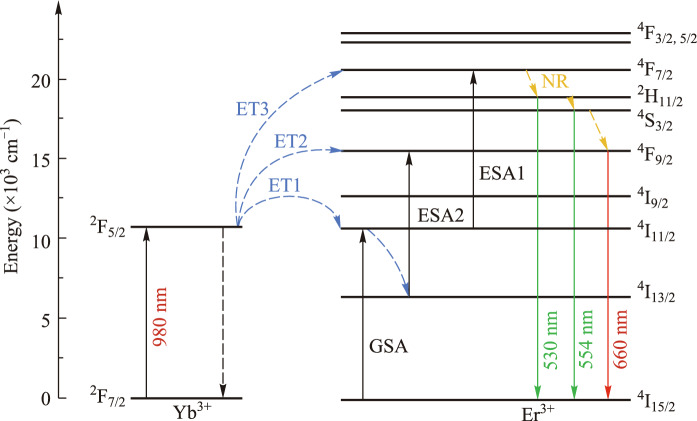
Simplified energy level diagram of Er^3+^ and Yb^3+^ ions and possible UC mechanisms involved in the sample (at low pump power density)

The simplified energy level diagrams of Er^3+^ and Yb^3+^ ions and possible luminescence processes at low pump power densities are illustrated in Fig. 5. The large absorption cross-section of Yb^3+^ ions in the near-infrared region and the amazing energy level overlap between Yb^3+^ and Er^3+^ ions result in the energy transfer (ET) process of Yb^3+^ to Er^3+^ ions, which primarily contributes to the generation of visible green and red light emission in the current work. Under the irradiation of a 980 nm laser, a large number of Yb^3+^ ions absorb near infrared photons and are excited from the ground state of ^2^F_7/2_ to the excited energy level of ^2^F_5/2_. Subsequently, the excited Yb^3+^ ions excite the neighboring Er^3+^ ions from the ^4^I_15/2_ ground state to the ^4^I_11/2_ energy level through an effective energy transfer process (ET1: ^2^F_5/2_ + ^4^I_15/2_ → ^2^F_7/2_ + ^4^I_11/2_). Additionally, Er^3+^ ions can directly absorb near-infrared photons through the ground state absorption process (GSA) and undergo a transition from the ^4^I_15/2_ ground state energy level to the ^4^I_11/2_ energy level. The adjacent Yb^3+^ ions excite a portion of the Er^3+^ ions located at the ^4^I_11/2_ level to the higher ^4^F_7/2_ level through the secondary energy transfer process (ET3: ^2^F_5/2_ + ^4^I_11/2_ → ^2^F_7/2_ + ^4^F_7/2_). Similarly, Er^3+^ ions can absorb a near-infrared photon through the excited state absorption process (ESA1), achieving the transition from the ^4^I_11/2_ to the ^4^F_7/2_ level. The ^4^F_7/2_ energy level undergoes nonradiative relaxation (NR) filling into the ^2^H_11/2_ and ^4^S_3/2_ energy levels. Ultimately, the transitions ^2^H_11/2_ → ^4^I_15/2_ and ^4^S_3/2_ → ^4^I_15/2_ result in green UC emission centered at 530 nm and 554 nm. The electrons in the ^2^H_11/2_/^4^S_3/2_ (Er^3+^) state can be partially non-radiatively relaxed to the ^4^F_9/2_ state (Er^3+^), and the ^4^F_9/2_ level can be filled directly from the ^4^S_3/2_ level. In addition, the electrons in the ^4^I_11/2_ can be depopulate by ET3, ESA1 and nonradiative to ^4^I_13/2_. Obviously, the electrons of the ^4^I_13/2_ energy level can be further excited to the ^4^F_9/2_ energy level through the excited state absorption process ESA2: ^4^I_13/2_ (Er^3+^) + a photon → ^4^F_9/2_ (Er^3+^), or through the energy transfer process (ET2) of the adjacent Yb^3+^ ions. Eventually, the radiative transition ^4^F_9/2_ → ^4^I_15/2_ occurs, resulting in the generation of red UC emission.

**Fig. 6:**
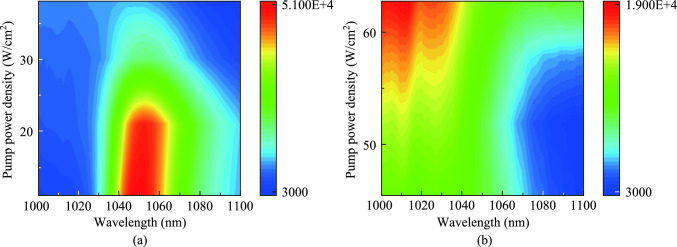
Contour plots of 5 at% Er^3+^:NaYb(MoO_4_)_2_ crystal upon the excitation of 980 nm laser with different pump power density. **a** At low pump power density. **b** At high pump power density

To directly explain the EBT process in the sample, we monitored the change of the emission intensity of Yb^3+^ ions under different power density excitation of 5 at% Er^3+^:NaYb(MoO_4_)_2_ crystal. Figure 6a gives the contour plot of the sample at low power density. The spectrum shows that the emission intensity of Yb^3+^ gradually decreases with the increase of power density, and the emission process of Yb^3+^ becomes weaker. As described in the energy transfer process of Fig. 5, this realizes the positive energy transfer from Yb^3+^ to Er^3+^ ions. In the case of high power density, as shown in Fig. 6b, it can be found that the very wide emission band of Yb^3+^ around 1um centered at 1048 nm is gradually weakened. In contrast, it shows an enhanced signal around 1010 nm. This is most likely due to the energy transfer of Er^3+^ to Yb^3+^ ions, that is, the possible EBT process occurs, resulting in the enhancement of Yb^3+^ signal. Finally, the yellow luminescence shown in Fig. 3b is realized.

**Fig. 7:**
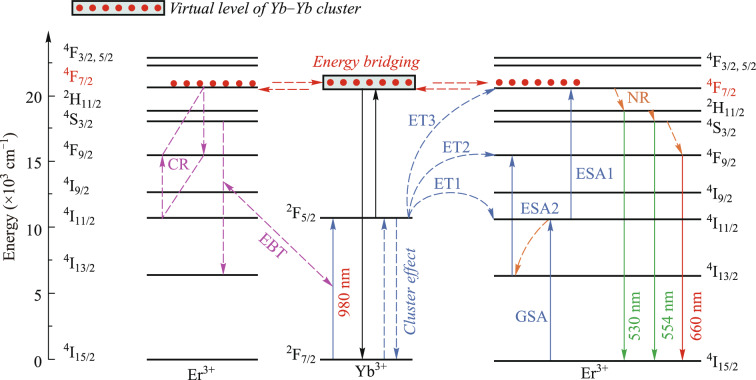
Simplified energy level diagram of Er^3+^ and Yb^3+^ ions and possible UC mechanisms involved in the sample (at high pump power density)

As shown in the contour plot of Fig. 5b, when the concentration of Yb^3+^ ions in the sample reaches a significant level and the pump power is high enough, it cannot be explained by the traditional UC mechanism of Fig. 5. The influence of other mechanisms on the luminescence of the sample should be considered, as shown in Fig. 7. At sufficient concentrations of Yb^3+^ ions, the red luminescence can be accounted for by the following two processes: the energy back transfer (EBT) process from Er^3+^ to Yb^3+^ (^4^S_3/2_ + ^2^F_7/2_ → ^4^I_13/2_ + ^2^F_5/2_) [5, 24, 25] and the cross-relaxation (CR) process between the Er^3+^ ions (^4^F_7/2_ + ^4^I_11/2_ → ^4^F_9/2_ + ^4^F_9/2_) [26, 27]. The EBT process can layout ^2^F_5/2_ energy levels and lowering the layout of the ^4^S_3/2_ energy level. Since the energy level difference between ^4^F_7/2_ and ^4^F_9/2_ (5200 cm^−1^) is comparable to the energy level difference between ^4^I_11/2_ and ^4^F_9/2_ (5100 cm^−1^), the CR process diminishes the intensity of green light emission [17, 28]. More importantly, the CR process becomes the dominant process only when the Er^3+^ ions concentration is high. Therefore, the EBT process is more efficient and dominates among the two processes [29], leading to the enhancement of red UC emission at high Yb^3+^ ion concentrations. This EBT-dominated mechanism leads to suppression of green UC luminescence and enhancement of red UC luminescence at higher Yb^3+^ ion concentrations, which is consistent with experimental observations. Notably, under high pump power excitation, it may be necessary to consider the energy exchange between two Er^3+^ ions. The highly filled ^4^F_7/2_ energy level reaches saturation, and any further energy contribution is transferred through the virtual energy level of the Yb–Yb cluster to the nearest ^4^F_7/2_ energy level of the other Er^3+^ ion, leading to a simultaneous transfer of energy through energy bridging between the two Er^3+^ ions [30].

## Conclusions

Overall, rare-earth ions doped NaYb(MoO_4_)_2_ phosphor and crystal, along with NaBi(MoO_4_)_2_ crystal, were successfully synthesized. The three samples exhibited two prominent green emission bands and a weak red emission band in the visible band. The luminescence images clearly show that the Er^3+^ ions doped Yb^3+^ ions self-activated NaYb(MoO_4_)_2_ phosphor and crystal exhibit yellow luminescence at high power density levels, attributed to the efficient EBT process (^4^S_3/2_ + ^2^F_7/2_ → ^4^I_13/2_ + ^2^F_5/2_). In contrast, the Yb^3+^/Er^3+^ ions codoped NaBi(MoO_4_)_2_ crystal displays bright green emission in the whole power density range. The findings indicate that the high Yb^3+^ ion concentration promotes an enhanced EBT process between Yb^3+^ and Er^3+^ ions, leading to the suppression of green emission and enhancement of red emission from Er^3+^ ions. In addition, crystals with long-range ordered structure exhibit weaker EBT process than phosphors with local ordered structure, a phenomenon possibly ascribed to their superior thermal stability. The relative intensity change of Yb^3+^ emission in 5 at%Er^3+^:NaYb(MoO_4_)_2_ crystal was further monitored, and the EBT process in self-activated samples at high power density was visually displayed. Based on the experimental results and comprehensive understanding of EBT mechanism, we provide a reliable direction for determining the optimal excitation power for optical temperature measurement.

## Data Availability

Data will be made available on request.
